# The effectiveness of a foot-care education program based on multi-theory model in diabetic patients with risk of foot ulceration: A randomized controlled trial protocol

**DOI:** 10.1371/journal.pone.0350892

**Published:** 2026-06-15

**Authors:** Huiwen Xu, Chen Wu, Lingyan Zhang, Yangqin Ju, Jie Chen, Qian Hu, Shuang Qiu

**Affiliations:** 1 School of Nursing, Faculty of Medicine, Yangzhou University, Yangzhou, China; 2 Faculty of Nursing, Nagano College of Nursing, Komagane, Japan; 3 Department of Orthopedics, Yangzhou Hospital of Traditional Chinese Medicine, Yangzhou, China; 4 Department of Nursing, Yangzhou Hospital of Traditional Chinese Medicine, Yangzhou, China; 5 Department of Endocrinology, Yangzhou Hospital of Traditional Chinese Medicine, Yangzhou, China; 6 Department of Neurology, Jiangsu Province Hospital of Chinese Medicine, Nanjing, China; 7 Department of Pharmacy, Yangzhou Hospital of Traditional Chinese Medicine, Yangzhou, China; University of Cape Town, SOUTH AFRICA

## Abstract

**Background:**

Diabetic foot ulcer (DFU) is one of the most common and serious complications of diabetes mellitus (DM). The high disability and fatality rates of DFU raise the importance of early medical treatment. However, delays in seeking medical treatment among DFU patients are common, emphasizing the need for targeted health education. This study aims to evaluate the effectiveness of a Multi-Theory Model (MTM)-based foot care education program on improving healthcare decision-making and foot care behaviors in diabetic patients with risk of foot ulceration.

**Method:**

A multicenter randomized controlled trial will be employed. Diabetic patients at risk of foot ulceration will be recruited from two different tertiary hospitals in different cities. Eligible participants will be randomly allocated to receive either routine education and a foot-care handbook (control group) or comprehensive foot-care education based on MTM (intervention group). The intervention period will last for 4 weeks, followed by a 3-month post-intervention follow-up. Questionnaire data and clinical data will be collected at baseline, immediately after the 4-week intervention, and at the 3-month follow-up. The primary outcome will be pre-hospital delay intentions. Foot care knowledge, foot care self-efficacy, and foot care behaviors will be investigated using questionnaires. Clinical data, including blood glucose, blood pressure, and body mass index (BMI), will also be assessed.

**Discussion:**

Based on the MTM, this study developed a comprehensive foot-care education program for diabetic patients at risk of foot ulceration. By integrating systematic, theory-driven educational components, the program may equips both hospitals and community health services with a structured approach to deliver targeted foot-care education.

**Trial registration The RCT registry number:** ChiCTR2400082853, 09/04/2024.

## Introduction

Diabetes mellitus (DM) is one of the most prevalent chronic diseases worldwide, posing a significant challenge to public health and has been a serious health problem. Epidemiological data from the International Diabetes Federation (IDF) indicates a global prevalence of 10.5% (536.6 million) among adults aged 20–79 in 2021, with a projected increase to 12.2% (783.2 million) by 2045 [1]. The clinical management of DM is complicated by the development of systemic sequelae, among which the diabetic foot ulcer (DFU) is one of the most severe. It is defined as an ulceration associated with neuropathy and/or peripheral arterial disease in the lower limb of a diabetic patient [2]. Every patient with DM has a 19–34% risk of developing a DFU at some point during their lifetime [3]. The global annual incidence is estimated to be between 9.1 and 26.1 million cases, highlighting its substantial burden on healthcare systems [1].

DFU poses a significant societal burden due to its substantial economic impact and severe clinical outcomes. Inadequate management leads to high costs for patients, families, and healthcare systems [4]. Clinically, approximately 20% of DFU patients undergo lower extremity amputation [5]. Moreover, DFU is associated with a significantly increased risk of mortality, with patients facing a 2.45 times higher risk of death compared to diabetic patients without foot ulcers [6].

The high mortality and disability rates associated with DFUs underscore the importance of prevention and seeking medical care promptly. Clinical guidelines suggest that patients should access specialized multidisciplinary care immediately after discovering an ulcer to decrease the likelihood of amputations and reduce the risk of mortality [7–10]. The National Institute for Health and Care Excellence (NICE) specifically advises that patients be referred to a multidisciplinary team within 24 hours of identifying a foot problem [11]. Nonetheless, a considerable number of patients fail to seek care during this critical window. Consequently, more than 50% of patients present with a diabetic foot infection (DFI) at their initial clinical visit, indicating a significant delay in obtaining the necessary medical intervention [12].

Substantial delays exist in the management of DFUs. The median time from symptom onset to specialist assessment ranges from 15 to 126 days, with a further median delay of 1–91 days from assessment to treatment initiation [13]. In China, this problem is severe, with pre-hospital delay rates ranging from 80.8% to 95.6% and a mean delay of 54.81 days before patients seek care [14]. A survey by Jun et al. [15] reported that among the diabetic patients who had foot problems in the past year, only 14.85% of them sought medical attention. Li et al. [16] found that only 33.2% of diabetic patients sought medical treatment within 1 week of DFU onset, while 48.7% saw a doctor within 2 weeks, with a median pre-hospital delay of 14 (range 1–354) days. These findings indicate substantial pre-hospital delays and prolonged visit times among patients with DFUs.

Delay in seeking medical assistance has been identified as an independent risk factor for poor outcomes among diabetic patients with limb complications [17]. It increases the risks of amputation and death [18]. Specifically, each 1 day delay in seeking medical care for DFUs is associated with a 3.5% increase in toe amputation (toe) rates [19]. As a result, it is suggested that a more aggressive and tailored education approach, which guides patients to act quickly in seeking medical care, is needed [20].

Evidence confirms that interventions integrating multiple theoretical approaches are more effective [21–23]. In this study, Multi-Theory Model (MTM) for health behavior change has been introduced. It overcomes the shortcomings of previous models by providing a dedicated framework for health behavior change and allowing for the study of long-term behavioral maintenance [24]. The MTM is structured in two components: behavior change initiation and sustenance. The initiation of behavior change is governed by three constructs: (1) participatory dialogue, where perceived benefits of a new behavior must outweigh its disadvantages; (2) behavioral confidence, or the belief in one’s capability to perform the behavior, derived from both internal and external sources; and (3) changes to the physical environment to provide tangible resources [24,25]. The sustenance of behavior change is similarly supported by three constructs: (1) emotional transformation, the ability to direct emotions toward a goal; (2) practice for change, which entails continuous, deliberate effort and adjustment of strategies; and (3) changes in the social environment, involving the creation of a supportive network, often facilitated by health educators.

This study will develop a foot-care education program based on MTM, and then employ a randomized controlled trial to evaluate its effectiveness among diabetic patients at risk of DFU.

### Aim and hypothesis

We aimed to investigate the effectiveness of an MTM-based foot-care education program in reducing patients’ pre-hospital delay intention and improving their foot-care knowledge, self-efficacy, and behaviors, as well as enhancing foot health and indicators including blood glucose and blood pressure.

It is hypothesised that, compared with those receiving foot-care handbook and routine education, the intervention group, who receive MTM-based foot-care education program, will have significantly strength in:

- Reducing pre-hospital delay intention.
- Improving foot-care knowledge, foot-care self-efficacy, and foot-care behavior.
- Improving foot health.
- Improving blood glucose and blood pressure.

## Methods

### Study design

This is a protocol of a randomized study in which a foot-care education program based on MTM will be implemented among diabetic patients at risk of foot ulceration. The study is an assessor-blined two-arm RCT. We used the SPIRIT guidelines to guide reporting of our trial protocol [26]. It has been registered in Chinese Clinical Trial Registry (No. ChiCTR2400082853).

### Location and setting

The study will be conducted at 2 different tertiary hospitals in Yangzhou and Nanjing, Jiangsu province.

### Participants and eligibility criteria

The study will recruit diabetic patients at risk of foot ulceration from a hospital setting. Participants must meet the World Health Organization diagnostic criteria for DM [27] and the risk criteria outlined in the Guidelines for the Diagnosis and Treatment of Diabetic Foot [28,29]. The inclusion criteria will be as follows: a) diagnosis of DM without an active foot ulcer, presenting with diabetic peripheral neuropathy (DPN), with or without foot deformity, peripheral artery disease, or a history of foot ulceration or amputation; b) age ≥ 18 years; c) possession and functional use of a smartphone with WeChat by both patient and caregiver; d) absence of communication-impairing conditions (e.g., hearing loss, aphasia, unconsciousness, psychiatric disorders); and e) provision of written informed consent. Exclusion criteria will include: a) severe concomitant conditions (renal impairment ≥ stage IV, cardiac function ≥ class III, or severe cerebrovascular disease); b) pregnancy or gestational diabetes; c) peripheral neuropathy attributable to non-diabetic causes (e.g., severe hepatic/renal disease, nutritional deficiencies, connective tissue disorders); and d) concurrent participation in another clinical trial.

### Sample size calculation and allocation

The sample size was calculated using G*Power 3.1 software for a two independent samples t-test. The primary outcome was pre-hospital delay intention (PHDI). A large effect size (Cohen’s d = 0.80) was adopted, which was justified by a previous study in patients with high-risk diabetic foot that reported a large effect size on pre-hospital delay intention [30]. The type I error rate (α) was set at 0.05 (two-tailed), and the statistical power (1 − β) was set at 0.85 [31]. The calculation yielded a required sample size of 30 participants per group. Accounting for an anticipated 15% attrition rate during follow-up, the final target sample size was set at 35 patients per group (total N = 70).

All the participants will be randomly divided into control group (n = 35) and intervention group (n = 35) upon admission. Randomization will be performed automatically by Microsoft Excel at enrollment. The “RAND ()” function in Microsoft Excel will be used to generate a series of random numbers by an independent researcher and kept in a sealed, opaque envelope. These numbers will be served as the basis for patient allocation into either the control group or intervention group.

### Blinding

The physician assessing outcome measures, as well as the statistician performing all statistical analysis will be blinded. The patients and the lead investigator in charge of the supervised training cannot be blinded for group allocation due to the nature of the intervention.

### Participant recruitment

To reach the target sample size efficiently, we will implement a focused recruitment strategy. Potential participants will be identified through electronic medical records at the participating clinics and approached during their regular visits. We will also use posters in waiting areas and collaborate with treating physicians for referrals.

### Intervention protocol

#### The control group: receive routine education and a hand book.

The control group will receive routine foot care education, which reflects the current standard of care in the participating clinical settings. During hospitalization, the nurses will conduct health education for patients. The content of health education will include daily self-care knowledge, such as regular exercise, blood glucose monitoring, medication, prevention and treatment of complications, foot care, etc. All patients will receive the Foot Care Guidance for Diabetic Patients handbook. It is developed referring to the domestic and international guidelines for diabetic foot prevention and management [32–34]. The handbook contains 3 parts regarding foot care for diabetic patients: the definition of diabetic foot (DF), the manifestation of DF, and the method of daily foot care.

Upon hospital discharge, the nurses will inform patients of precautions when leaving the hospital, instruct them on medication-taking methods, emphasize the importance of self-care at home, educate them to pay attention to healthy eating, regular exercise, taking medicine as prescribed by the doctor, strengthening daily foot care, advise patients to seek medical attention promptly if their condition worsens or they experience any unusual discomfort at home.

After a patient discharge, the nurses will follow up with the patient via telephone once a month, for a total of 4 follow-up telephone interviews. The follow-up will include assessing the patient’s blood glucose control and foot care. Additionally, the research team will provide guidance on disease-related issues the patient encountered at home.

#### The intervention group: receive MTM-based foot-care education.

When a patient is admitted to hospital, they will receive the same routine nursing as the control group. In addition, they will be invited to participate in the MTM-based foot-care education program.

### Participatory dialogue

Participatory dialogue will be developed through one-on-one face-to-face communication. On day 1–3 of admission, one-on-one communication will be conducted by a trained senior nurse. We will initiate discussions by encouraging patients to share their routine practices of examining their feet. Patients will be prompted to describe their understanding of their current foot condition, and they will be asked to detail the specific preventive foot care practices they have independently implemented. Based on the knowledge of the patients’ condition and education, the nurses will explain the clinical manifestations and severity of DF, consequences of DF, the disadvantages of delayed medical treatment. The duration of each education session will be 10–20 minutes, using the *Foot Care Guidance for Diabetic Patients* handbook to complete the explanation. To further improve the patient’s cognition of DF, the nurses will discuss the benefits as well as barriers of foot care with the participants.

### Behavioral confidence

For the “behavioral confidence” construct, health education lectures will be held by the research team who have been specifically trained. The education session will be delivered to participants with PowerPoint and video presentation for about one hour in the clinical ward, along with their family members or carers. On days 4–7 of admission, the nurses will demonstrate and instruct the patients on proper foot care. The ‘teach-back’ method will be introduced to improve the patients’ comprehension [35].

### Changes in the physical environment

Regarding “Changes in the physical environment”, we will provide participants with a bag of foot-care supplies, which includes a cotton towel, a pair of pressure-relief insoles, an infrared thermometer, a toenail file and samples of Vaseline. These supplies are all necessary for foot care. The research nurse will provide verbal persuasion as she shows the patient each item of foot care supplies and review the purpose of each.

In addition, we will also develop a mobile application (app) to provide a software support system for the patients. The smartphone app for diabetes management (register number: 2018SR446465) consists of 4 modules: syndrome differentiation, body differentiation and health preservation, thesaurus, and interactive follow-up. Patients in the intervention group will be required to install the software before hospital discharge. In addition, the research nurses will teach the participants to use the app correctly and ensure that they can use it by themselves.

### Emotional transformation

The intervention features a multi-sensory simulation of the DF experience, which aligns with the MTM’s “emotional transformation” framework. Participants will undergo a standardized protocol where a 2 kg sandbag is attached to their ankles to mimic symptoms of lower extremity arterial disease, and sponge platforms will be used to induce neuropathic sensory differences. This immersive activity is designed to promote emotional engagement with potential foot complications.

Post-discharge support will be sustained via a dedicated WeChat platform, including: 1) a public account for structured educational content, and 2) a communication group for peer interaction and consultation with the research team. Additional one-to-one follow-ups will provide personalized psychological support and address individual patient concerns. The specific content pushed via the WeChat public account is detailed in Table 1.

**Table 1:** Overview of the foot-care education program.

Module		Time	Methods	Content
**Initiation of Behavior Change**	Participatory Dialogue	Days 1–3 of admission	One-on-one face-to-face communication	1.1 Clinical manifestations and severity of diabetic foot
				1.2 Consequences of diabetic foot
				1.3 Disadvantages of delayed medical treatment for diabetic foot problems
				1.4 Benefits of foot care
				1.5 Barriers to foot care
	Behavioral Confidence	Days 4–7 of admission	Health education lecture Teach-back method	2.1 Foot care steps
				2.2 Foot massage
				2.3 Foot exercises
				2.4 Foot care precautions
	Changes in the Physical Environment	One day before discharge	Provide foot-care supplies	3.1 Foot-care supplies: a towel, a pair of pressure-relief insole, an infrared thermometer, toenail file, and samples of Vaseline.
			App installation	3.2 App installation and usage support
**Sustenance of Behavior Change**	Emotional Transformation	One day before discharge	Experiential education	4.1.1 Symptom experience of lower extremity arterial disease
				4.1.2 Podiatric symptom experience
		After hospital discharge	Create a WeChat official account Send the tweets to the WeChat group	4.2.1 What is diabetic foot?
				4.2.2 Causes of diabetic foot
				4.2.3 Risk factors for diabetic foot
				4.2.4 Clinical manifestations of diabetic foot
				4.2.5 Prevention of diabetic foot
				4.2.6 Diagnosis of diabetic foot
				4.2.7 Self-examination of foot at home
				4.2.8 Daily foot care
				4.2.9 Foot massage and exercises
				4.2.10 Nail care and footwear selection
				4.2.11 Seeking medical help
				4.2.12 Blood glucose management
				4.2.13 Nutrition management for diabetes
				4.2.14 Medication management
				4.2.15 Insulin injection
				4.2.16 Physical exercise
				4.2.17 Psychological adjustment
	Practice for Change	After discharge, once a week	Keep a record WeChat contact	5.1 Keep a record on *Foot Care Diary Card*
				5.2 WeChat reminder
	Changes in the Social Environment	After discharge	Family support Social support	6.1 Family engagement: WeChat group for collaborative foot care.
				6.2 WeChat consultation.
				6.3 Home visit: foot examination and foot care education.

### Practice for change

Regarding “Practice for change”, a *Foot Care Diary Card* will be designed and distribute to the patients to record their performance of each specific foot care behavior every day, and the research team will be responsible for contacting the participants through WeChat to improve adherence to intervention protocols. There will be a clear instruction on the card teaching the patients daily foot care behaviors, including inspecting feet, washing and drying feet, applying lotion to their feet, foot massage, and foot exercises. The patients will receive a weekly follow-up telephone call or WeChat reminder from the nurses encouraging them to adhere to foot care practices.

### Changes in the social environment

“Changes in the social environment” will be developed by tele-nursing and home visits. After discharge, the family members, friends or the primary caregivers will be invited to join in the WeChat communication group, and they could consult the doctors and nurses in the group. In addition, the researcher nurse will also provide some information regarding foot care for them via WeChat. Tele-nursing support is available throughout the 4-week intervention and follow-up. Additional home visits will be provided if required.

In order to ensure the consistent delivery of the program, we will develop a program training for research nurses. Table 1 shows the overview of the foot-care education program.

### Patient and public involvement

During the design of this study, patient input was sought to improve the program. Three target patients reviewed the initial draft. Their perspectives on the feasibility and burden of study procedures helped to finalize the protocol.

### Data collection and management

Following randomization, baseline data will be collected via researcher-administered questionnaires, physical examinations, and biochemical tests. To ensure data quality at this stage, research nurses will check completed questionnaires for completeness immediately after collection.

A key strategy to promote participant retention and complete follow-up involves maintaining regular contact. Participants will receive reminder calls or messages prior to each follow-up assessment. Additionally, we will offer flexible scheduling for follow-up visits and express appreciation for their continued participation. For participants who discontinue the assigned intervention, we will seek their permission to continue collecting some outcome data from their medical records to enable an intention-to-treat analysis.

All paper-based forms will be transcribed into a dedicated electronic database. A comprehensive data management plan will be implemented to ensure data integrity and security. This will include double-data entry for key variables to minimize errors, along with automated range checks for data values to identify outliers. The final dataset will be anonymized and stored on a secure, password-protected computer with regular backups, accessible only to authorized research personnel.

To ensure the validity of the trial and the safety of participants, all patients are allowed to continue their regular diabetic management and must seek immediate care for any acute foot problems. However, participants are not permitted to join other structured foot care programs or trials throughout the duration of this study. The use of newly prescribed specialized footwear for ulcer prevention is prohibited unless it is deemed medically necessary, in which case it must be documented. All such concurrent care is recorded to evaluate its potential impact on the outcomes.

The results will be submitted for publication in peer-reviewed international journals and presented at relevant conferences.

### Outcome measures

**Fig 1:**
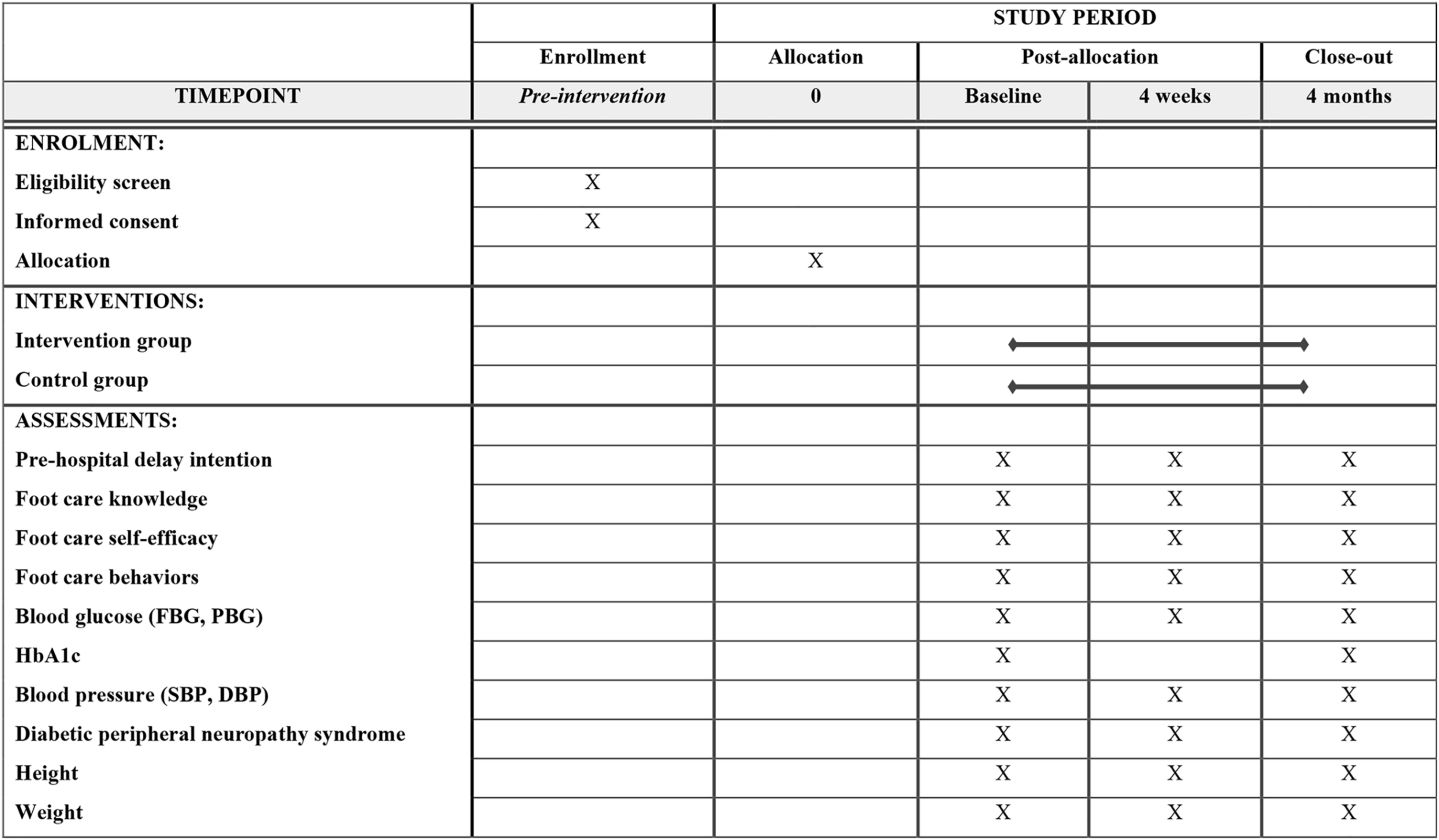
Schedule of enrolment, interventions, and assessments. No., number; DFU, Diabetic foot ulcer; FBG, Fasting blood glucose, PBG, post-prandial blood glucose; SBP, systolic blood pressure; DBP, diastolic blood pressure; HbA1c, glycated hemoglobin A1c.

Schedule of enrolment, interventions, and assessments are presented in Fig 1. Baseline (T 0) data will be collected right after participants’ eligibility is determined and consent signed. Before and after completing 4 weeks intervention (T1), patients’ pre-hospital delay intentions, foot care knowledge, foot care self-efficacy, foot care behaviors, blood glucose, blood pressure, weight, and DPN syndrome, will be assessed.

A supplementary 3-month follow-up assessment (T2) will be conducted outside the primary RCT framework. This evaluation will replicate the baseline measurements for all outcome variables and will additionally document the incidence of DFU as a safety outcome.

### Primary outcome

#### Pre-hospital Delay Intention Survey (PHDI).

The PHDI scale, developed by Xu et al. [36], serves as a validated predictor of delay behavior in diabetic patients at risk of foot ulceration [37]. This instrument comprises seven items assessed on a 4-point Likert scale, yielding total scores between 7 and 28. Higher scores indicate stronger delay intentions. The scale demonstrates good internal consistency, with a Cronbach’s α of 0.884 [36].

### Secondary outcome

#### Audit of Diabetes Knowledge Survey (ADKnowl).

Foot-care knowledge will be assessed using the relevant dimension of the Chinese version of the ADKnowl questionnaire, which was modified and validated by Zhu [38]. This version has demonstrated strong reliability (Cronbach’s α = 0.909) and validity (content validity index = 0.923). Consistent with prior research [39], the foot-care subscale was utilized, encompassing 24 items across six domains: foot examination, daily care, toenail trimming, foot treatment, shoe selection, and skin treatment. Responses are recorded as true, false, or ‘don’t know,’ with a total possible score of 24. Higher scores indicate superior foot-care knowledge.

#### Diabetic Foot Care Self-Efficacy Scale (DSES).

Foot-care self-efficacy will be assessed using the corresponding subscale from the Chinese version of the DSES. This instrument was originally developed by Hurley and Shea [40], subsequently translated and modified by Wang [41], and validated in mainland China by Wan et al. [42], demonstrating strong reliability (Cronbach’s α = 0.91). The 5-item foot-care subscale evaluates confidence in performing key behaviors such as daily foot examination, proper footwear selection, and nail care. Items are rated on a 5-point Likert scale from “definitely not” (1 point) to “definitely yes” (5 points), yielding total scores between 5 and 25, with higher scores indicating greater self-efficacy. This focused application of the foot-care component follows established methodological precedent [39].

#### Nottingham Assessment of Functional Foot-care Questionnaire (NAFF).

Foot self-care practices will be assessed using the Chinese version of the NAFF, developed by Lincoln [43] and translated by Li et al. [44]. This 24-item instrument evaluates five behavioral domains: daily inspection, hygiene, protection, appropriate footwear, and help-seeking for foot problems. Responses are scored on a 0–3 frequency scale, with eight reverse-scored items yielding a total score range of 0–72. Higher scores indicate better self-care practices. The Chinese version demonstrates adequate reliability (Cronbach’s α = 0.77, test-retest reliability = 0.74).

#### Toronto Clinical Scoring System (TCSS).

The TCSS will be employed to assess DPN. This validated instrument combines evaluation of sensory perception, deep tendon reflexes, and neuropathic symptoms, generating a continuous score from 0 to 19 points [45]. A trained research nurse will administer the assessment using a standardized peripheral nerve testing toolkit.

### Blood glucose

Glycemic control parameters will be prospectively collected through standardized clinical procedures. Venous blood samples will be obtained after a minimum eight-hour fast to measure fasting blood glucose (FBG), postprandial blood glucose (PBG), and glycated hemoglobin (HbA1c) through biochemical analysis, with results extracted from clinical records.

### Physical examination

Physical examinations will be performed by trained research nurses using standardized procedures. Participants’ weight and height will be measured using a digital weighing scale and a stadiometer, respectively. Blood pressure measurements will record systolic (SBP) and diastolic (DBP) values using a sphygmomanometer. DFU will be also assessed.

### Statistical analyses

All statistical analyses will be performed using SPSS version 27.0 (IBM Corp., Armonk, NY, USA). Continuous variables with a normal distribution will be expressed as mean and standard deviation, while those with a non-normal distribution will be presented as median and interquartile range. Categorical variables will be summarized as frequencies and percentages and compared using the chi-square test or Fisher’s exact test as appropriate.

Longitudinal changes in primary and secondary outcomes measured at baseline, one month post-intervention, and three months post-intervention will be assessed using repeated-measures analysis of variance. The raw outcome value measured at each time point will serve as the dependent variable. Group will be treated as the between-subjects factor, and time as the within-subjects factor.

**Fig 2:**
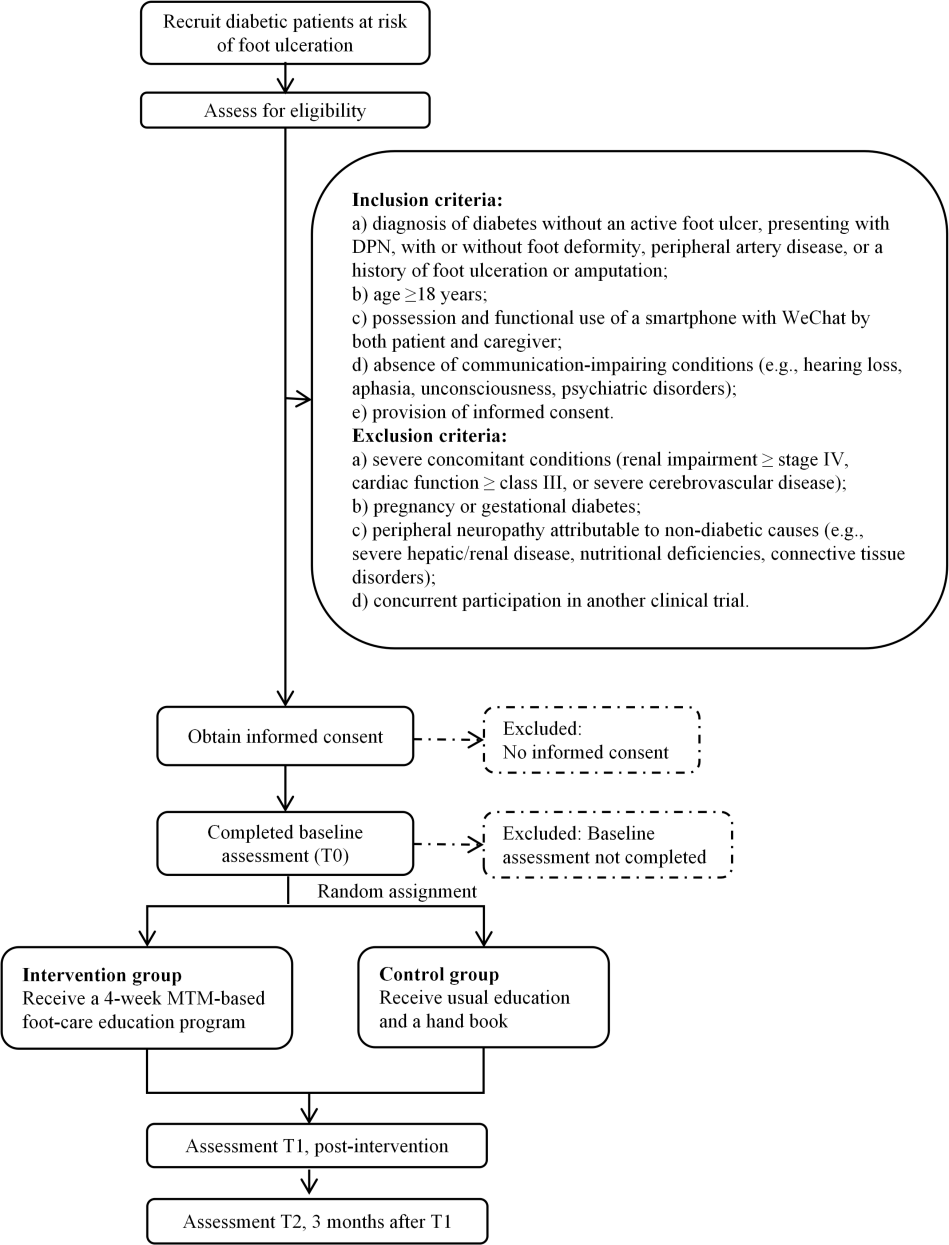
Flow chart of the RCT study protocol.

For secondary outcomes, the Bonferroni method will be applied to adjust P-values for multiple comparisons to control the type I error rate. Both intention-to-treat and per-protocol analyses will be performed. For the intention-to-treat analysis, missing data will be handled using multiple imputation under the missing at random assumption, with 20 imputed datasets generated and results pooled according to Rubin’s rules. A sensitivity analysis using complete case analysis will be conducted under the missing not at random assumption to assess the stability of the findings. Statistical significance will be defined as a two-sided adjusted P-value of less than 0.05. The study flowchart is presented in Fig 2.

### Ethic consideration

This study will be conducted in accordance with the principles of the Declaration of Helsinki. The protocol has been approved by the Ethics Committee of the School of Nursing at Yangzhou University (Approval No. YZUHL20230118).

The intervention is classified as minimal risk, as it involves no invasive procedures and its content aligns with established clinical guidelines. Three specific potential risks have been identified. First, participants may experience anxiety due to heightened awareness of foot ulcer risk; psychological support and counseling will be available. Second, there is a possibility of misunderstanding medical advice; this will be mitigated by using pre-tested educational materials and conducting post-session confirmation of understanding. Third, behavioral overcorrection may occur; moderation education and follow-up monitoring will be implemented to promote safe self-care practices.

All identifiable participant information will be replaced with a unique study code to ensure anonymization. Electronic data will be stored on a password-protected computer with access restricted to authorized personnel, and regular backups will be performed to prevent data loss. Consent forms and case report forms will be kept in locked filing cabinets within a secure research office. Access to identifiable information will be limited to the principal investigator and designated research nurses involved in participant follow-up and clinical care coordination; no other team members will have access to such data.

Participants will receive a compensation of 30 RMB per person to cover time and travel expenses. This amount is consistent with the study budget and local standards for minimal-risk research. In the event of accidental injury resulting from research procedures, necessary medical care and related expenses will be provided, along with appropriate economic compensation in accordance with relevant laws and regulations in China.

If a participant develops an active foot ulcer during the trial period, a standardized management procedure will be followed. The participant will be referred to a diabetic foot specialist within 24 hours. Intervention sessions will be temporarily paused, although the participant will remain included in the intention-to-treat analysis. Weekly follow-up will be conducted, and the event will be reported as a serious adverse event to the ethics committee within 72 hours.

Written informed consent will be obtained from all participants after a detailed explanation of the study’s purpose and significance. Participants will be explicitly informed that participation is voluntary and that they have the unconditional right to withdraw at any time. The control group will receive the equivalent intervention upon study completion.

### Reports the status and timeline of the study

The study is currently in the pre-recruitment phase. Participant recruitment is scheduled to commence on February 1, 2026, and is expected to be completed by May 31, 2026. Data collection is planned to conclude by December 2026. Final results are anticipated to be available by March 2027, following data cleaning, statistical analysis, and interpretation.

## Discussion

DFU continues to pose a significant global health challenge, profoundly affecting patients’ quality of life, the utilization of medical resources, and economic burdens. The decision to seek treatment for DFU involves a complex medical process, which includes accurately identifying DFU symptoms, determining the need for specialized medical care, and ultimately taking appropriate action to seek emergency medical treatment. Failure at any of these stages can lead to pre-hospital delays and worsened outcomes, including increased risks of amputation and mortality, and missed opportunities for life-saving treatments. These delays are often attributed to patients’ lack of awareness and understanding of the severity of DFU, resulting in significant knowledge gaps that frequently lead to delayed medical consultation. For example, a systematic review by Novita et al. highlighted that patients with DFU often lack the necessary self-care strategies and motivation to manage their wounds effectively, leading to prolonged healing times and increased risk of complications. These findings highlighted the urgent need for comprehensive education and support to improve patients’ understanding and management of DFU, thereby reducing pre-hospital delays and improving patient outcomes [46].

Health education is important in mitigating pre-hospital delays among patients with DF, as it enhances illness perception and foot care practices [47,48]. However, traditional interventions often rely on a single theory, which may not address the complex behavioral processes required for effective foot care and timely medical consultation. In contrast, the MTM-based intervention in this study offers a more comprehensive framework by integrating multiple health behavior theories, thereby overcoming the limitations of single-theory interventions. This comprehensive approach ensures sustained behavior change through digital platforms and social networks, which is crucial for preventing DFU and reducing pre-hospital delays. As the fourth-generation models, MTM has individual, social, and environmental applications in developing plans for educational interventions, which is an appropriate framework for developing an education program. To our knowledge, it has not been previously implemented in the population of diabetic patients at risk of foot ulceration.

This study represents the first randomized, parallel-controlled, multicenter trial of foot health education based on the MTM for diabetic patients at risk of foot ulceration within the context of Chinese culture, offering comprehensive and detailed data. This innovative approach uniquely integrates multiple health behavior theories, addressing the complex interplay of emotional, physical, and social factors, which is crucial for initiating and sustaining health behavior change. The MTM-based foot care program comprehensively considers the complex interplay of emotional, physical, and social factors, and is expected to reduce pre-hospital delays and prevent DFU by enhancing patients’ foot care knowledge, foot care self-efficacy, and foot care behaviors. However, this study has several limitations. First, the sample may not be fully representative of all diabetic patients in China due to regional and socioeconomic variations. Second, the intervention’s reliance on self-reported outcomes might introduce recall bias. Lastly, the relatively short follow-up period may limit the assessment of long-term behavioral changes and DFU incidence.

## Supporting information

S1 File SPIRIT checklist. (DOCX)

## Abbreviations

DFU: Diabetic foot ulcer
DM: Diabetes mellitus
MTM: Multi-theory model
BMI: Body mass index
IDF: International Diabetes Federation
DF: Diabetic foot
DPN: Diabetic peripheral neuropathy
WHO: World Health Organization
IWGDF: International Working Group on the Diabetic Foot
TCM: Traditional Chinese Medicine
TCSS: Toronto Clinical Scoring System
PHDI: Pre-hospital Delay Intention Survey
ADKnowl: Audit of Diabetes Knowledge Survey
DSES: Diabetic Foot Care Self-Efficacy Scale
NAFF: Nottingham Assessment of Functional Foot-care Questionnaire
FBG: Fasting blood glucose
PBG: Post-prandial blood glucose
SBP: Systolic blood pressure
DBP: Diastolic blood pressure
HbA1c: Glycated hemoglobin A1c
